# The Role of Intraoperative Indocyanine Green Fluorescence Angiography in Reducing Anastomotic Leak After Colorectal Resection: A Systematic Review of Randomised Controlled Trials

**DOI:** 10.7759/cureus.117951

**Published:** 2026-10-15

**Authors:** Layan Abu-Khalaf, Yahia Khalil, Raya Abu-Khalaf, Sandra Abu-Khalaf, Humaid AlKaabi

**Affiliations:** 1 Cardiology, Blackpool Victoria Hospital, Blackpool, GBR; 2 Internal Medicine, St Mary's Hospital, Isle of Wight, GBR; 3 Medicine, Royal College of Surgeons in Ireland, Dublin, IRL; 4 Gastroenterology, University Hospital Limerick, Limerick, IRL; 5 General Practice, Zayed Military Hospital, Abu Dhabi, ARE

**Keywords:** anastomotic leak, colorectal resection, fluorescence angiography, indocyanine green, near-infrared imaging, perfusion assessment

## Abstract

Anastomotic leak (AL) has a reported incidence of 3%-19% after colorectal resection. The aim of this Preferred Reporting Items of Systematic Reviews and Meta-analyses (PRISMA) 2020 compliant systematic review is to determine whether intraoperative indocyanine green fluorescence angiography (ICG-FA) reduces clinically significant anastomotic leak and improves peri-operative outcomes compared with standard white-light assessment in adults undergoing colorectal resection with primary anastomosis. We searched MEDLINE, Embase, Cochrane Central Register of Controlled Trials (CENTRAL), and Web of Science from inception to March 2026. Eligible study designs included randomised controlled trials (RCTs) enrolling adults undergoing elective colorectal resection with primary anastomosis and comparing intra-operative ICG-FA with standard white-light assessment. The primary outcome was “clinically relevant anastomotic leak” as defined by the International Study Group of Rectal Cancer. Six RCTs enrolling 4,366 randomised patients, of whom 4,221 were evaluable: FLAG (n=377), De Nardi et al. (n=240), EssentiAL (n=839), AVOID (n=931), ICG-COLORAL (n=1,136), and IntAct (n=698). EssentiAL was the only trial to demonstrate a statistically significant reduction in all-grade AL (7.6% (32/422) vs 11.8% (49/417); relative risk (RR) 0.645; p=0.041) and grade B/C AL (4.7% (20/422) vs 8.2% (34/417); p=0.044). AVOID, ICG-COLORAL, and IntAct did not meet their primary endpoints but consistently favoured left-sided resections in subgroup analyses. IntAct showed an absolute five percentage-point reduction in grade B/C AL (10% (36/343) vs 15% (54/355); adjusted odds ratio (OR) 0.667; 95% confidence interval (CI) 0.419-1.060; p=0.087). No serious ICG-related adverse events were reported. Grading of Recommendations Assessment, Development, and Evaluation (GRADE) certainty of evidence for the primary outcome was moderate. Current RCT evidence does not support routine ICG-FA across all colorectal anastomoses. Leak rates are most consistently reduced for left-sided and low rectal resections where inferior mesenteric artery (IMA) ligation may compromise anastomotic perfusion.

## Introduction and background

Anastomotic leak (AL) remains the most serious complication of colorectal resection. Reported incidence ranges from 3% to 19% [1], varying by anastomotic site and patient factors, and despite surgical advances and improved perioperative care, the incidence figure has remained unchanged [2]. The consequences of AL include: prolonged inpatient admissions, emergency operation for end stoma, delayed adjuvant chemo-radiotherapy, and poorer overall cancer-specific survival rates [3,4].

The standard intraoperative approach to perfusion assessment relies on white-light evaluation of bowel colour, arterial bleeding from the transected bowel end, and mesenteric vessel pulsation [5]. However, these measures are subjective, lighting-dependent, and individually calibrated to the surgeon's experience. Studies have shown poor predictive accuracy of white light assessment for AL [1,6].

Indocyanine green (ICG) is an intravenous dye with an established safety profile. It is administered through a bolus injection, where it binds to plasma proteins and emits near-infrared fluorescence detectable at 830 nm by modern laparoscopic and robotic imaging systems, enabling real-time perfusion visualisation [5].

Clinical data for ICG fluorescence angiography (ICG-FA) emerged with observational studies. PILLAR II (Perfusion Assessment in Laparoscopic Left-Sided/Anterior Resection), a multi-institutional prospective series of 139 laparoscopic lower anterior resections (LAR), reported an AL rate of 1.4%, far lower than the 10%-12% historical benchmark [7]. PILLAR III (Perfusion Assessment in Left-Sided/Low Anterior Resection), a subsequent randomised trial, which enrolled 347 patients undergoing LAR, did not show a statistically significant reduction in AL with ICG compared with standard assessment [8].

Recent randomised controlled trials (RCTs) between 2020 and 2025, including FLAG (A Study of Perfusion of Colorectal Anastomosis Using Fluorescence Angiography), De Nardi et al., EssentiAL (Blood Perfusion Assessment by Indocyanine Green Fluorescence Imaging for Minimally Invasive Rectal Cancer Surgery), AVOID (Anastomotic Leakage and Value Of Indocyanine Green in Decreasing Leakage Rates), ICG-COLORAL (Indocyanine Green Fluorescence Imaging in Prevention of Colorectal Anastomotic Leakage), and IntAct (Intra-operative fluorescence angiography to prevent anastomotic leak in rectal cancer surgery) [3-6,9,10], have reported inconsistent findings. Some trials reported a significant reduction in anastomotic leak, whereas others showed no statistically significant benefit overall. Subgroups did suggest benefit in left-sided and rectal resections. This review aims to synthesise data from eligible RCTs to determine whether ICG-FA reduces AL after colorectal anastomoses.

## Review

Methods

This systematic review was conducted and reported in accordance with the Preferred Reporting Items for Systematic Reviews and Meta-Analyses (PRISMA) 2020 guidelines [11]. A protocol was developed prior to database searching.

Eligibility Criteria

Eligibility criteria were defined using the population, intervention, comparator, outcome (PICO) framework and are summarised in Table 1.

**Table 1:** PICO eligibility criteria for study inclusion Eligibility criteria were defined a priori and applied at both title/abstract and full-text screening. ISGRC grade B denotes a leak requiring active therapeutic intervention without relaparotomy, and grade C a leak requiring reoperation [12]. AL=Anastomotic leak; HAR=high anterior resection; ICG=indocyanine green; ICG-FA=indocyanine green fluorescence angiography; ISGRC=International Study Group of Rectal Cancer; LAR=low anterior resection; PICO=population, intervention, comparator, outcome; RCT=randomised controlled trial.

Domain	Inclusion Criteria
Population	Adults aged ≥18 years undergoing elective colorectal resection with primary anastomosis, including: right and left hemicolectomy, sigmoid colectomy, Higher Anterior Resection (HAR), LAR, subtotal colectomy
Intervention	Intraoperative ICG-FA for anastomotic perfusion assessment, using any dose or timing protocol
Comparator	Standard white light visual assessment without ICG
Primary Outcome	Clinically significant AL, defined as a defect in intestinal wall integrity requiring active therapeutic intervention, corresponding to ISGRC grade B or C [12], within 30 or 90 postoperative days.
Secondary Outcomes	re-operation incidence, length of hospital stay, change in intraoperative plan, postoperative complication rate, and ICG-related adverse outcomes
Study design	RCTs

Search Strategy and Study Selection

The following electronic databases were searched: MEDLINE, Embase, Cochrane Central Register of Controlled Trials (CENTRAL), and Web of Science Core Collection from database inception to March 2026. The Search strategies combined MeSH and Emtree controlled vocabulary terms: Anastomosis, Surgical," "Colorectal Surgery," "Indocyanine Green," and "Anastomotic Leak" with free-text terms: colorectal anastomosis, ICG fluorescence angiography, and anastomotic leak. English-language studies were filtered during the search. The full search strategy is provided in the Appendices. After removing duplicate records, two screeners independently reviewed titles and abstracts against the inclusion criteria. Screened articles were listed using Microsoft Excel (Microsoft, Redmond, WA), including their full-text link and reference, and were tagged with “included”, “excluded” and “uncertain”. A third reviewer resolved disagreements.

Data Extraction 

Two reviewers independently extracted data using a piloted standardised extraction form. Fields included study author and year, country, design, number of centres, recruitment period, sample size by arm, patient demographics, surgical indication, procedure type, operative approach, anastomotic site, ICG dose and timing, imaging platform, comparator description, verbatim leak definition, follow-up duration, AL events by arm, reoperation by arm, length of hospital stay (LOS), intraoperative plan change counts, complication rates, ICG-related adverse events, effect estimates with covariates, funding sources, and declared conflicts of interest. Where both adjusted and unadjusted estimates were reported, adjusted figures were used, as these account for potential confounding.

Risk of Bias Assessment

Two reviewers individually assessed risk of bias for the primary outcome of anastomotic leak in each RCT using the Cochrane risk of bias (RoB 2) tool, evaluating five domains: randomisation process, deviations from intended interventions, missing outcome data, outcome measurement, and selection of reported results. Each domain was flagged with either: low risk, some concern, or high risk, with written justification. Disagreements were resolved by a third author. 

Synthesis and GRADE Assessment

Due to heterogeneity in enrolled populations, ICG-FA protocols, and anastomotic leak definitions, a meta-analysis was not undertaken. The findings were synthesised narratively by study design and, among RCTs, further grouped by anastomotic site: rectal/rectosigmoid, colorectal, and colonic excluding LAR. The Grading of Recommendations Assessment, Development, and Evaluation (GRADE) framework was used to assess certainty of the RCT evidence for the primary outcome (AL) by considering risk of bias, inconsistency, indirectness, imprecision, and publication bias.

Results

Study Selection

The database identified 1,847 records. After de-duplication, 1,412 records underwent title and abstract screening; 1,298 were excluded. 114 remaining articles were assessed for eligibility, and 108 were excluded (lacked a comparator group, reported perfusion surrogate outcomes only, excluded study type, represented duplicate cohorts, or were not available in English). Six eligible RCTs were therefore included. Figure 1 shows the PRISMA 2020 flow diagram (Figure 1). 

**Figure 1:**
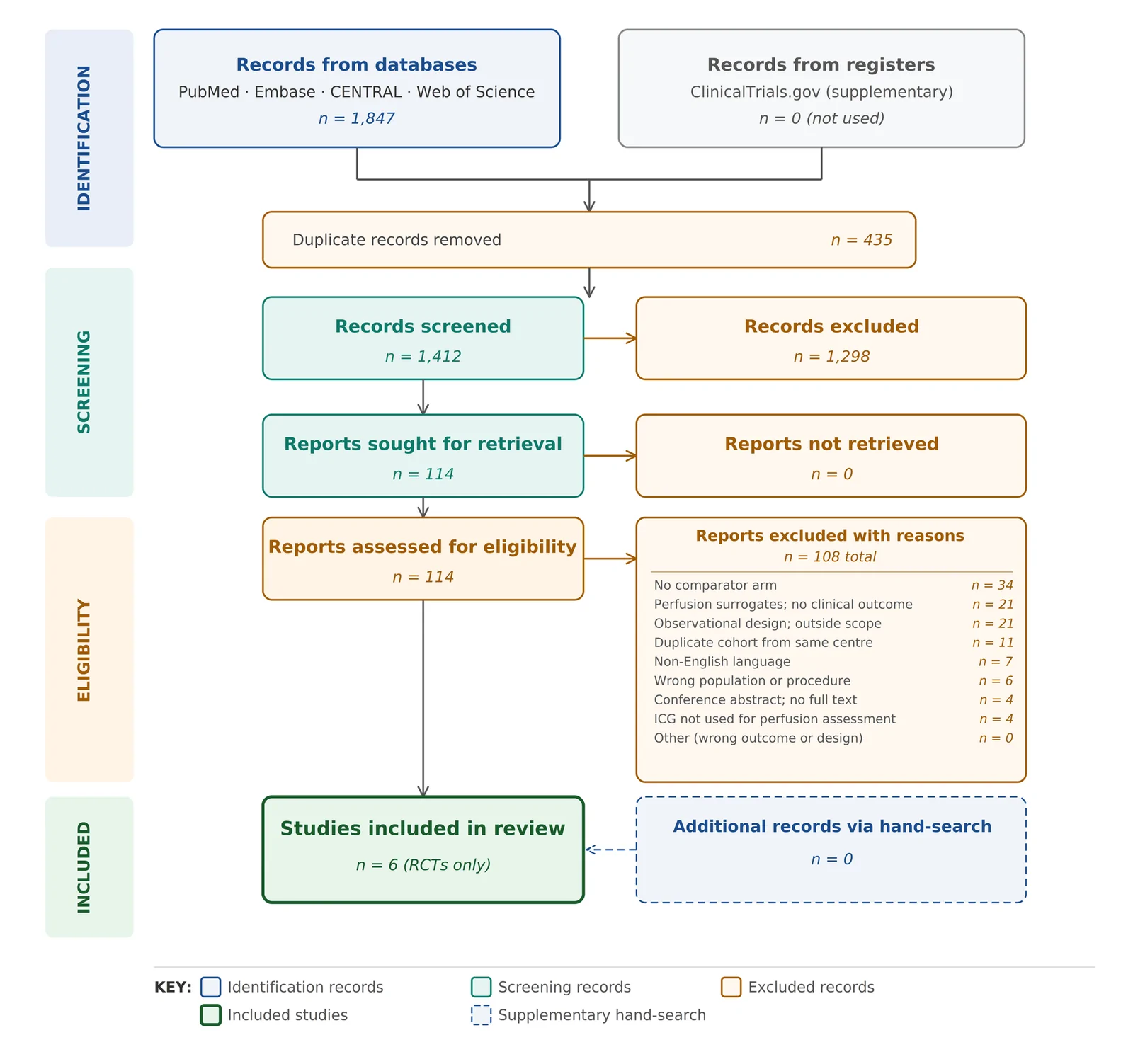
PRISMA 2020 Flow Diagram Records were identified from PubMed, Embase, CENTRAL, and Web of Science from database inception to March 2026. After 435 duplicate records were removed, 1,412 records were screened and 114 full-text reports were assessed for eligibility, of which 108 were excluded for the reasons shown. Six randomised controlled trials met the eligibility criteria. Trial registers were searched as a supplementary source and hand-searching identified no additional eligible records. Diagram adapted from the PRISMA 2020 statement [11]. CENTRAL=Cochrane Central Register of Controlled Trials; ICG=indocyanine green; PRISMA=Preferred Reporting Items for Systematic Reviews and Meta-Analyses; RCT=randomised controlled trial.

Overview of Included RCTs 

Across the six RCTs, 4,366 patients were randomised, of whom 4,221 were included in the primary outcome (Table 2). Trials were published between 2020 and 2025 across Italy, Japan, Russia, the Netherlands, Finland, and eight European countries [4]. FLAG [9], EssentiAL [3], and IntAct [4] enrolled rectal and rectosigmoid populations. However, De Nardi et al. [10] predominantly enrolled patients of left-sided colonic and rectal resections. AVOID [6] enrolled all patients undergoing minimally invasive colorectal resection. ICG-COLORAL [5] excluded LAR cohorts, only including right- and left-sided colonic surgery. Primary outcome follow-up was 30 days in four trials [3,5,9,10] and 90 days in two [4,6]. Four trials [3-6] classified leaks using the International Study Group of Rectal Cancer (ISGRC) grading system. However, FLAG [9] and De Nardi et al. [10] reported AL as a binary clinical outcome without using severity-graded classification.

**Table 2:** Characteristics of Included Randomised Controlled Trials AV=Anal verge; HAR=high anterior resection; LAR=low anterior resection; MIS=minimally invasive surgery.

Trial & Year	Setting (N) (ICG/Control)	Population	Comparator	ICG Protocol & Follow-up
FLAG [9] (2020)	Russia (1 centre) 187/190	Sigmoid & rectal Ca; open/MIS	White light	0.2 mg/kg (single dose) 30 days
De Nardi et al. [10] (2020)	Italy (3 centres) 118/122	Lap left-sided & rectal (2–15 cm AV)	White light	0.3 mg/kg (dual dose) 30 days
EssentiAL [3] (2023)	Japan (41 centres) 422/417	Rectal Ca (<12 cm AV); MIS	White light	5 mg fixed (dual dose) 30 days
AVOID [6] (2024)	Netherlands (8 centres) 463/468	All MIS colorectal (~20% LAR)	White light	5 mg fixed (pre-anastomosis) 90 days
ICG-COLORAL [5] (2025)	Finland (5 centres) 567/569	Lap colorectal (LAR excluded)	White light	5 mg fixed (dual dose) 30 days
IntAct [4] (2025)	Europe (28 centres) 343/355	Rectal Ca (≤15 cm AV); MIS	White light	0.1 mg/kg (dual dose) 90 days

Primary Outcome: Anastomotic Leak by Trial

*FLAG trial (Alekseev et al., 2020) *[9]*:* FLAG was a single-centre RCT in Moscow which enrolled 380 patients. After excluding three post randomisation, 377 patients with sigmoid or rectal cancer undergoing stapled colorectal anastomosis were included, randomised 1:1 to ICG-FA or standard visual assessment. AL occurred in 9.1% (17/187) of the ICG-FA group versus 16.3% (31/190) of controls (p=0.04) [9]. On subgroup analysis by anastomotic height, the reduction was confined to low anastomoses (4-8 cm from the anal verge): 14.4% versus 25.7% (p=0.04). However, for higher anastomoses, no significant difference was observed (1.3% vs 4.6%; p=0.37) [9]. No ICG-related adverse events were reported. 

*De Nardi et al. (2020) *[10]:* *This three-centre Italian RCT enrolled 252 patients undergoing laparoscopic left-sided colonic or rectal resection. After 12 post randomisation withdrawals, 240 patients were analysed: ICG-FA (n=118) or standard assessment (n=122) [10]. Perfusion was assessed twice: before and after anastomosis formation. ICG angiography identified insufficient perfusion in 13/118 cases, prompting further proximal bowel resection (11%) [10]. AL occurred in 5% (6/118) of ICG patients versus 9% (11/122) of controls (p=0.2) [10]. The trial was underpowered; the observed control AL rate fell below that assumed in the power calculation, limiting the ability to detect a statistically significant difference. ICG-FA was concluded to be safe and feasible, though a significant reduction in AL was not demonstrated [10].

*EssentiAL Trial (Watanabe et al., 2023) *[3]*: *EssentiAL was a multi-centre phase III RCT conducted in Japan, which enrolled 850 patients with stage 0-III rectal cancer, all scheduled for minimally invasive rectal sphincter-preserving surgery. Of 839 eligible patients, 422 were analysed in the ICG arm and 417 in the control arm [3].

All-grade AL (ISGRC grades A+B+C) occurred in 7.6% (32/422) of ICG patients versus 11.8% (49/417) of controls (relative risk (RR) 0.645; 95% confidence interval (CI) 0.422-0.987; p=0.041) [3]. Grade B/C AL was 4.7% (20/422) versus 8.2% (34/417) (p=0.044), and reoperation was 0.5% (2/422) versus 2.4% (10/417) (p=0.021) [3]. The trial demonstrated a statistically significant reduction in AL, though the observed 4.2 percentage-point difference fell short of the pre-specified six percentage-point threshold. EssentiAL was the only ISGRC-graded trial in this review to report a statistically significant reduction in AL, with 30-day follow-up and no serious ICG-related adverse events recorded [3].

*AVOID trial (Faber et al., 2024)*[6]*: *AVOID was a multicentre RCT conducted in the Netherlands. In this trial, 982 patients were enrolled, and after exclusion, 931 patients were analysed, undergoing laparoscopic or robotic colorectal resections with primary anastomosis: 463 in the ICG arm and 468 in the control arm [6]. The primary outcome was ISGRC grade B/C AL within 90 days [6,12].

The primary endpoint was not met. Grade B/C AL occurred in 7% (32/463) of ICG patients versus 9% (42/468) of controls (p=0.24) [6]. However, a pre-specified subgroup analysis of left-sided resections (RR 0.59; 95%CI 0.35-1.00, p=0.047) and a post-hoc analysis of rectosigmoid resections (RR 0.58; 95% CI 0.34-1.00, p=0.045) both demonstrated significant reductions in AL with ICG-FA [6].

*ICG-COLORAL trial (Rinne et al., 2025) *[5]*: *ICG-COLORAL enrolled 1,136 patients undergoing laparoscopic colorectal resection across five Finnish centres, with LAR excluded throughout [5]. ICG was given as a fixed 5 mg intravenous dose, administered twice per case [5].

Overall AL occurred in 5.8% (33/567) of ICG patients versus 7.9% (45/569) of controls (odds ratio (OR) 0.73; 95% confidence interval (CI) 0.48-1.13; p=0.16), which did not reach statistical significance [5]. In left-sided operations, AL was 5.2% (14/267) with ICG versus 9.5% (23/243) without (OR 0.55; 95% CI 0.29-1.05; NS), suggesting a more pronounced benefit in operations where high inferior mesenteric artery (IMA) ligation places marginal perfusion at risk [5]. No ICG-related adverse events were reported [5].

*IntAct trial (Jayne et al., 2025) *[4]*: *IntAct enrolled patients with rectal cancer (lower tumour margin ≤15 cm from the anal verge) undergoing laparoscopic or robotic anterior resection at 28 centres across eight European countries between October 2017 and August 2023 [4,13]. ICG was given intravenously at 0.1 mg/kg after rectal mobilisation and again following anastomosis formation [4]. Of 766 randomised patients, 698 were included in the primary analysis (343 ICG, 355 control) [4].

Grade B/C AL within 90 days occurred in 10% (36/343) of ICG patients versus 15% (54/355) of controls (adjusted OR 0.667; 95% CI 0.419-1.060; p=0.087), which did not reach statistical significance [4]. When broken down by severity, grade A leaks occurred in 3% (11/343) of ICG patients versus 6% (20/355) of controls, grade B in 3% (11/343) versus 9% (31/355), and grade C in 7% (25/343) versus 6% (23/355) [4]. ICG appeared to reduce lower-severity leaks, while grade C rates - those requiring re-operation - were similar between arms [4]. The trial fell short of its planned 880 patients due to COVID-19, leaving it underpowered against its original design [4,13]. The primary outcome definitions, leak rates by arm, and effect estimates for all six trials are summarised in Table 3.

**Table 3:** Primary Outcome Results of Included RCTs AL=Anastomotic leak; AV=anal verge; ISGRC=International Study Group of Rectal Cancer; LAR=low anterior resection; NS=not significant; OR=odds ratio; RR=relative risk.

Trial	AL Definition	AL: ICG arm	AL: Control arm	Effect estimate	p value
FLAG [9]	Clinical/radiological (ungraded)	9.1% (17/187) overall; low anastomosis subgroup 14.4%	16.3% (31/190) overall; low anastomosis subgroup 25.7%	Significant overall and in low anastomosis subgroup (4–8 cm AV)	0.04 (overall)
De Nardi et al. [10]	All-grade (ungraded)	5% (6/118)	9% (11/122)	Numerical trend favouring ICG; NS	0.2
EssentiAL [3]	All-grade A+B+C (ISGRC [12])	7.6% (32/422)	11.8% (49/417)	RR 0.645 (95% CI 0.422–0.987)	0.041
EssentiAL [3]	Grade B+C (ISGRC [12])	4.7% (20/422)	8.2% (34/417)	Significant; reoperation also reduced (0.5% vs 2.4%)	0.044
AVOID [6]	Grade B+C (ISGRC [12]), 90 days	7% (32/463)	9% (42/468)	NS overall; AL occurred in 7% of ICG patients versus 9% of controls (p = 0.24) ]; left-sided: RR 0.59 (95% CI 0.35–1.00), p = 0.047 (pre-specified); rectosigmoid: RR 0.58 (95% CI 0.34–1.00), p = 0.045 (post-hoc [6],	0.24
ICG-COLORAL [5]	All-grade; LAR excluded	Overall: 5.8% (33/567) Left-sided: 5.2% (14/267)	Overall: 7.9% (45/569) Left-sided: 9.5% (23/243)	Overall: OR 0.73 (95% CI 0.48–1.13); NS Left-sided: OR 0.55 (95% CI 0.29–1.05); NS	NS (overall and left-sided subgroup)
IntAct [4]	Grade B+C (ISGRC [12]), 90 days	10.5% (36/343)	15.2% (54/355)	Adj OR 0.667 (95% CI 0.419–1.060)	0.087

Intraoperative Change of Operative Plan

Across the included trials, ICG-FA prompted surgeons to revise planned transection margins in a clinically relevant proportion of cases: 19.3% (36/187) in FLAG [9,14], 11% (13/118) in De Nardi et al. [10], and 9% (41/463) in AVOID [6]. This is consistent with the broader literature; McEntee et al. reported a pooled plan change rate of 8.4% (385/4570) across 34 comparative studies of ICG-FA in colorectal surgery [15]. In these cases, standard visual assessment alone would have proceeded with an anastomosis at a site ICG identified as poorly perfused.

Meta-analyses of largely observational data report lower AL rates when the transection point was changed based on ICG findings versus when no change was made [16], and McEntee et al. found ICG-FA was associated with a significant reduction in AL across comparative studies [15]. Patients whose transection point was revised tended to be older, have higher BMI, greater neoadjuvant therapy exposure, and lower tumours, with their final anastomoses sitting higher and low anterior resection syndrome (LARS) scores numerically lower at six months [15]. These are observational associations and should not be interpreted as evidence of causal benefit without randomised confirmation [15].

Secondary Outcomes

*Reoperation*: EssentiAL was the only trial to demonstrate a statistically significant reduction in reoperation rate (0.5% (2/422) vs 2.4% (10/417); p=0.021 [3]. AVOID [6] and IntAct [4] trended in the same direction without reaching significance. No trial reported a higher reoperation rate in the ICG arm [3-6,9,10].

*Length of hospital stay*: LOS was reported inconsistently across trials, and no RCT demonstrated a significant difference between the groups. 

*Safety*: No serious ICG-related adverse events were reported across any of the six trials. 

Risk of Bias Assessment

Table 4 presents risk-of-bias results using RoB 2. No trial was rated to have a high overall risk of bias. All six were judged to have some concerns, largely driven by performance bias: an unavoidable limitation in open surgical trials where the operating team necessarily knows which arm they are in. AVOID raised additional concerns around potential under-ascertainment of radiological leaks, given the absence of mandatory post-operative contrast enema [6]. IntAct raised concerns about COVID-19-driven recruitment modifications, though these were openly reported [4]. EssentiAL and ICG-COLORAL had the strongest randomisation and allocation concealment procedures and were rated low risk across most individual domains [3,5].

**Table 4:** Risk of Bias Assessment (RoB 2) for Included RCTs ¹Performance bias: operating team necessarily unmasked to allocation in all trials - inherent to open surgical RCTs. ²No standardised quantitative ICG interpretation criteria; reliance on subjective surgeon assessment. ³Absence of mandatory post-operative contrast enema may have underascertained radiological grade A leaks. ⁴COVID-19-related recruitment modifications; transparently reported in the primary publication. D=Domain; RoB 2=Cochrane Risk of Bias tool version 2.

Trial	D1: Randomisation	D2: Deviations	D3: Missing data	D4: Measurement	D5: Reporting	Overall
FLAG [9]	Low	Some concerns¹	Low	Some concerns²	Low	Some concerns
De Nardi et al. [10]	Low	Some concerns¹	Low	Some concerns²	Low	Some concerns
EssentiAL [3]	Low	Some concerns¹	Low	Low	Low	Some concerns
AVOID [6]	Low	Some concerns¹	Low	Some concerns³	Low	Some concerns
ICG-COLORAL [5]	Low	Some concerns¹	Low	Low	Low	Some concerns
IntAct [4]	Low	Some concerns¹,⁴	Low	Low	Low	Some concerns

GRADE Assessment

For the primary outcome of clinically significant AL, defined as ISGRC grade B/C or equivalent, the overall certainty of evidence was rated as moderate. Risk of bias was not downgraded, as no included trial was judged to be at serious or high overall risk. Inconsistency was downgraded by one level because trial findings were not uniform: EssentiAL demonstrated a significant reduction in AL, whereas AVOID, ICG-COLORAL, and IntAct did not. This may reflect differences in enrolled populations, anastomotic site, ICG-FA protocols, and outcome definitions. Indirectness was not downgraded, as the included trials directly addressed the review question. Imprecision was not downgraded given the overall sample size, with variation in effect estimates considered under inconsistency. Publication bias was not strongly suspected, as three included studies were large multicentre RCTs with neutral findings, although formal assessment was limited by the small number of included RCTs.

Discussion

The principal finding of this systematic review is that ICG-FA does not reduce AL consistently across all colorectal anastomoses. The evidence appears more favourable in left-sided colonic and low rectal resections, where high IMA ligation may compromise anastomotic blood supply. A null result in mixed or all-colorectal populations does not necessarily rule out a meaningful benefit in higher-risk cases.

Why EssentiAL [3] met its primary endpoint while AVOID [6], ICG-COLORAL [5], and IntAct [4] did not is best explained by differences in the patients enrolled. EssentiAL recruited exclusively patients with rectal cancer undergoing minimally invasive sphincter-preserving surgery; a group at high baseline AL risk, where the anastomosis relies heavily on marginal perfusion after IMA ligation [3]. AVOID cast a wider net, enrolling patients across all resection sites and both benign and malignant indications [6]. Including right hemicolectomies, where the ileocolic pedicle is preserved and perfusion is rarely at risk, diluted any benefit in the higher-risk left-sided anastomoses. This likely explains the null overall result and aligns with the significant reduction seen in the left-sided rectosigmoid subgroup [6].

ICG-COLORAL specifically examined non-rectal colonic surgery by excluding LAR entirely to address evidence gaps [5]. The left-sided subgroup result (OR 0.55; 95% CI 0.29-1.05) points to the left-sided benefit hypothesis but fell short of statistical significance in a trial powered for an overall non-LAR population [5]. This is hypothesis-supporting rather than confirmatory.

IntAct enrolled patients with treatment plans directly relevant to European practice; those with rectal cancer undergoing curative anterior resection at specialist centres [4,13]. The trial did not meet its primary endpoint (adjusted OR 0.667; 95% CI 0.419-1.060; p=0.087) [4]. Recruitment fell short of the planned 880 patients, reaching only 766 due to COVID-19 disruption, leaving the trial underpowered relative to its original design [4,13]. The adjusted OR of 0.667 represents a 33% relative reduction in grade B/C AL with a five percentage-point absolute difference; clinically non-trivial, even without statistical significance. The grade breakdown is telling: ICG-FA appeared to reduce grade A leaks (3% (11/343) vs 6% (20/355)) and grade B leaks (3% (11/343) vs 9% (31/355)), while grade C rates - those requiring reoperation - were similar between arms (7% (25/343) vs 6% (23/355)) [4]. This suggests ICG-FA may prevent lower-severity perfusion-related leaks without substantially reducing the risk of the most severe anastomotic failures, which are likely determined by factors beyond perfusion alone.

Intraoperative plan change rates of 19.3% (36/187) and 11% (13/118) were reported in FLAG [9,14], and De Nardi et al. [10], each representing cases where white-light assessment alone would have proceeded with an anastomosis at a site ICG identified as poorly perfused. This does not automatically translate into fewer leaks, as anastomotic healing depends on more than perfusion alone. Considerable variation in how ICG was applied across trials, including differences in ICG dose, number of injections, timing, and imaging platform, limits direct cross-trial comparison and may partly explain the inconsistent results observed.

The Society of American Gastrointestinal and Endoscopic Surgeons (SAGES) 2025 guidelines recommend ICG for left-sided colorectal anastomosis with moderate certainty of evidence, based on RCT and observational data [14]. This systematic review is broadly consistent with this recommendation. The FLUOCOL-01 (Intraoperative Indocyanine Green Fluorescence Angiography in Colorectal Surgery to Prevent Anastomotic Leakage) trial, targeting left-sided intraperitoneal anastomoses as its primary population, is expected to provide randomised evidence in this subgroup [17]. Formal health economic evaluation of ICG-FA falls outside the scope of this review.

Several meta-analyses have shown consistent evidence favouring ICG-FA use in left-sided and rectal resections. Kazi et al. reviewed six RCTs of left-sided anastomosis and found a pooled OR of 0.586 (95% CI 0.434-0.792), an absolute AL reduction of 4.7%, and no heterogeneity (I²=0%). The study concluded that ICG could warrant consideration as standard of care in this setting [18]. Lucarini et al. found that in rectal resections exclusively, AL rates were reduced from 13.9% without ICG to 9.0% with ICG (p<0.003) [19]. Balaphas et al. had a broader analysis, including eight RCTs enrolling 3,999 patients, confirming a significant protective effect against AL (OR 0.64; 95% CI 0.39-0.98; I²=0%), with an absolute risk reduction of four percentage points [20]. These three analyses showed directional findings consistent with this review.

Mixed design and observational studies reported larger effect sizes than RCT evidence. Tang et al. and Shen et al. reported pooled odds ratios of 0.46 and 0.27, respectively [21,22], representing 54% and 73% lower odds of AL compared with controls. This shows more favourable evidence for ICG-FA than the RCT-level estimates observed in this review. Similarly, Watanabe et al.’s cohort study in laparoscopic LAR reported significantly lower odds of AL with ICG-FA (OR 0.427; p=0.042), meaning patients in the ICG-FA group had 57% lower odds of developing a clinically significant leak [23]. The effect sizes are consistently larger amongst the observational studies, which can be due to selection bias. Hospitals adopting ICG-FA tend to be specialist centres with higher candidate volumes, making them achieve better outcomes through surgical experience, team expertise, and patient selection, independent of ICG-FA use itself. These factors can independently influence AL risk. Randomisation reduces this confounding bias, allowing RCT estimates in this review to be considerably more modest. Afridi et al.’s meta-analysis restricted its data to eight RCTs contributing to AL outcome. 4,047 patients were enrolled, and a statistically significant 34% reduction in AL was shown with ICG (RR 0.66; 95% CI 0.54-0.81; p<0.0001; I²=0%) [24]. No statistical heterogeneity was detected across these RCTs (I²=0%), suggesting a consistent effect at the pooled RCT level.

Two recent reviews and meta-analyses suggest strong RCT-level evidence for ICG-FA in colorectal surgery. Maroli et al. included 10 RCTs enrolling 4,885 patients, and results showed a significant reduction in AL (OR 0.64; p<0.0001) [25]. The subgroup analysis had similar findings to this review, showing benefit in left colon (p=0.003) and rectal resections (p=0.0002), with no significant benefit in right colonic resections (p=0.94) [25]. Ryan et al. [26] pooled RCT data into a meta-analysis, meta-regression and trial sequential analysis. The primary outcome risk ratio was 0.66 (95% CI 0.56-0.78; p<0.0001), with a number needed to treat (NNT) of 24, consistent with Afridi et al. [24]. Ryan et al.'s trial sequential analysis is particularly relevant as it confirmed that enough patients have been enrolled to draw a reliable conclusion; the accrued sample exceeded the required information size of 2,183 patients, supporting the reliability of the overall finding [26]. Future work should focus more on which resection site would benefit from ICG-FA. This review showed that benefit is most consistent in left-sided and rectal resections, with one trial meeting its endpoint, pointing towards selective use as consistent with current meta-analytic data.

Strengths and limitations

This review incorporates the most recent phase III RCT evidence in this field, including IntAct [4] and ICG-COLORAL [5]; two large trials published in 2025 that were unavailable to many earlier systematic reviews in this field. The six included RCTs span multiple countries, range from single-centre to 41-site trials, enrol over 4,000 randomised patients, and cover the full range of colorectal anastomotic sites from right-sided colonic to low rectal resections, providing a breadth of randomised evidence that no single trial achieves alone.

Focusing exclusively on RCTs rather than pooling randomised and observational data strengthens internal validity by reducing susceptibility to the confounding and selection bias that may inflate treatment effects in cohort analyses. Independent dual-reviewer processes supported methodological rigour for screening, data extraction, and risk of bias assessment, alongside a formal GRADE appraisal of the primary outcome. The review was conducted and reported in accordance with PRISMA 2020 [11].

The limitations of this review include restricting searches to English-language publications, which may have excluded evidence relevant to our search criteria from other languages. Meta-analysis was not undertaken given substantial heterogeneity across trials in patient populations, ICG protocols, leak definitions, and follow-up duration, which are differences that would make interpreting pooled estimates difficult.

At the trial level, FLAG [9] was single-centre and used an ungraded leak definition, limiting direct comparison with ISGRC-graded trials. De Nardi et al. [10] was substantially underpowered, as the AL rate of 9% was much lower than what the power calculation assumed, meaning the trial lacked sufficient events to achieve statistical significance despite a four percentage point difference between arms. As for AVOID [6], the absence of mandatory post-operative contrast enema raises the possibility of under-ascertainment of radiological leaks. IntAct [4] fell short of its planned sample size due to COVID-19 disruption and may therefore have been underpowered to detect the pre-specified effect. ICG-COLORAL [5] excluded LAR entirely, limiting how findings can be applied to rectal surgery, an area where AL risk is highest. The lack of standardisation across all trials, including ICG dosing, timing, and fluorescence interpretation criteria, introduced variability that complicates cross-trial inference. Finally, this review relied on published aggregate data rather than individual participant data. This review was not prospectively registered with PROSPERO prior to commencement, which is acknowledged as a limitation.

## Conclusions

The RCT evidence included in this review does not support routine ICG-FA across all colorectal anastomoses. Trials with right-sided and mixed colorectal populations have not shown a statistically significant reduction in anastomotic leak. The evidence is more favourable in left-sided and low rectal resections, although not uniformly statistically significant. EssentiAL was the only trial to report a statistically significant reduction in both all-grade and clinically relevant AL, while AVOID, ICG-COLORAL, and IntAct reported reductions in the same direction without reaching statistical significance. Subgroup analyses in left-sided resections consistently favoured AL reduction. No serious ICG-FA adverse events were reported across the included trials, and the technique prompted intraoperative revision of the planned transection margin in 19.3% (36/187) and 11% (13/118) of cases in FLAG and De Nardi et al., respectively - operations where white-light assessment alone would not have identified that the resection margins are at a poorly perfused site. The overall certainty of evidence, rated by GRADE, was moderate.

The evidence points towards selective rather than routine adoption, specifically in left-sided and rectal resections where high IMA ligation places anastomotic perfusion at risk. Subgroup findings across this review are exploratory and need confirmatory trial data before they can guide practice as definitive evidence. Future research should include adequately powered trials in anatomically defined high-risk anastomoses, standardisation of ICG dosing, clear criteria for interpreting fluorescence perfusion, defining adequate perfusion, and health economic analysis.

## Appendices

Full electronic search strategies

The following search strategies were applied in MEDLINE via PubMed, Embase via Embase.com, CENTRAL, and Web of Science Core Collection from database inception to March 2026. Both high-sensitivity and high-specificity searches were applied in each database. An English-language filter was applied at retrieval. 

MEDLINE via PubMed

1a. High-Sensitivity Search Limits: Humans; English language; inception to March 2026.

("Anastomosis, Surgical"[Mesh] OR "Colorectal Surgery"[Mesh] OR "colorectal anastomosis"[tiab] OR "colorectal resection"[tiab] OR "rectal resection"[tiab] OR "sigmoid resection"[tiab] OR colectomy[tiab] OR proctectomy[tiab] OR "anterior resection"[tiab] OR "low anterior resection"[tiab] OR anastomosis[tiab] OR anastomoses[tiab])

AND

("Indocyanine Green"[Mesh] OR "indocyanine green"[tiab] OR ICG[tiab] OR "fluorescence angiography"[tiab] OR "near-infrared fluorescence"[tiab] OR NIRF[tiab] OR "fluorescence imaging"[tiab] OR "fluorescence-guided"[tiab])

AND

("Anastomotic Leak"[Mesh] OR "anastomotic leak"[tiab] OR "anastomotic leakage"[tiab] OR "anastomotic failure"[tiab] OR "anastomotic dehiscence"[tiab])

1b. High-Specificity Search Limits: Humans; English language; inception to March 2026.

("Anastomosis, Surgical"[Mesh] OR "colorectal anastomosis"[tiab] OR "colorectal resection"[tiab] OR "rectal resection"[tiab] OR "sigmoid resection"[tiab] OR colectomy[tiab] OR "low anterior resection"[tiab] OR anastomosis[tiab])

AND

("Indocyanine Green"[Mesh] OR "indocyanine green"[tiab] OR ICG[tiab] OR "fluorescence angiography"[tiab] OR "near-infrared fluorescence"[tiab])

AND

("anastomotic leak"[tiab] OR "anastomotic leakage"[tiab] OR "anastomotic failure"[tiab] OR "anastomotic dehiscence"[tiab])

AND

(randomized[tiab] OR randomised[tiab] OR trial[pt] OR trial[tiab])

Embase via Embase.com

2a. High-Sensitivity Search Limits: English language; inception to March 2026.

('anastomosis'/exp OR ('colorectal anastomosis':ti,ab OR 'colorectal resection':ti,ab OR 'rectal resection':ti,ab OR 'sigmoid resection':ti,ab OR colectomy:ti,ab OR 'low anterior resection':ti,ab OR anastomosis:ti,ab))

AND

('indocyanine green'/exp OR ('indocyanine green':ti, ab OR ICG: ti,ab OR 'fluorescence angiography':ti,ab OR 'near-infrared fluorescence':ti, ab OR NIRF:ti,ab))

AND

('anastomotic leak'/exp OR ('anastomotic leak':ti, ab OR 'anastomotic leakage':ti,ab OR 'anastomotic failure':ti,ab OR 'anastomotic dehiscence':ti,ab))

2b. High-Specificity Search Limits: English language; inception to March 2026.

('anastomosis'/exp OR ('colorectal anastomosis':ti,ab OR 'colorectal resection':ti,ab OR 'rectal resection':ti,ab OR 'sigmoid resection':ti,ab OR colectomy:ti,ab OR 'low anterior resection':ti,ab))

AND

('indocyanine green'/exp OR ('indocyanine green':ti,ab OR ICG:ti,ab OR 'fluorescence angiography':ti,ab OR 'near-infrared fluorescence':ti,ab))

AND

('anastomotic leak':ti,ab OR 'anastomotic leakage':ti,ab OR 'anastomotic failure':ti,ab OR 'anastomotic dehiscence':ti,ab)

AND

(randomized:ti,ab OR randomised:ti,ab OR 'randomized controlled trial'/de OR trial:ti,ab)

3. Cochrane Central Register of Controlled Trials (CENTRAL)

3a. High-Sensitivity Search Limits: English language; no date limits.

(colorectal OR "colorectal anastomosis" OR "colorectal resection" OR "rectal resection" OR "sigmoid resection" OR colectomy OR "low anterior resection" OR anastomosis) AND ("indocyanine green" OR ICG OR "fluorescence angiography" OR "near-infrared fluorescence" OR NIRF) AND ("anastomotic leak" OR "anastomotic leakage" OR "anastomotic failure" OR "anastomotic dehiscence")

3b. High-Specificity Trial-Focused search Limits: English language; inception to March 2026. 

("colorectal anastomosis" OR "colorectal resection" OR colectomy OR "low anterior resection" OR anastomosis) AND ("indocyanine green" OR ICG OR "fluorescence angiography" OR "near-infrared fluorescence") AND ("anastomotic leak" OR "anastomotic leakage" OR "anastomotic dehiscence" OR "anastomotic failure") AND (randomized OR randomised OR trial OR RCT)

Web of Science Core Collection

4a. High-Sensitivity search Refined at retrieval: Document Type = Article; Language = English; no date limits.

TS=("colorectal anastomosis" OR "colorectal resection" OR "rectal resection" OR "sigmoid resection" OR colectomy OR "low anterior resection" OR anastomosis) AND TS=("indocyanine green" OR ICG OR "fluorescence angiography" OR "near-infrared fluorescence" OR NIRF) AND TS=("anastomotic leak" OR "anastomotic leakage" OR "anastomotic dehiscence" OR "anastomotic failure")

4b. High-Specificity search Refined at retrieval: Document Type = Article; Language = English; no date limits.

TS=("colorectal anastomosis" OR "colorectal resection" OR colectomy OR "low anterior resection" OR anastomosis) AND TS=("indocyanine green" OR ICG OR "fluorescence angiography") AND TS=("anastomotic leak" OR "anastomotic leakage" OR "anastomotic dehiscence" OR "anastomotic failure") AND TS=(randomized OR randomised OR trial OR RCT)
